# Adults of Sun Coral *Tubastraea coccinea* (Lesson 1829) Are Resistant to New Antifouling Biocides

**DOI:** 10.3390/toxics12010044

**Published:** 2024-01-06

**Authors:** Isabela Martins, Kátia Cristina Cruz Capel, Denis Moledo de Souza Abessa

**Affiliations:** 1 Biosciences Institute, Campus of Rio Claro, São Paulo State University—UNESP, Avenida 24A, 1515, Rio Claro 13506-900, SP, Brazil; isabela.martins1@unesp.br; 2 National Museum, Federal University of Rio de Janeiro, Quinta da Boa Vista, São Cristóvão, Rio de Janeiro 20940-040, RJ, Brazil; katiacapel7@gmail.com; 3 Centre of Marine Biology, University of São São Paulo (CEBIMar/USP), Rodovia Doutor Manoel Hipólito do Rego, km. 131,5, Pitangueiras, São Sebastião 11612-109, SP, Brazil; 4 Biosciences Institute, Campus of São Vicente, São Paulo State University—UNESP, Praça Infante Dom Henrique, s/n, São Vicente 11330-900, SP, Brazil

**Keywords:** *Tubastraea coccinea*, antifouling, DCOIT, nanomaterials, ecotoxicology

## Abstract

Biocides used in antifouling (AF) paints, such as 4,5-dichlorine-2-n-octyl-4-isothiazole-3-one (DCOIT), can gradually leach into the environment. Some AF compounds can persist in the marine environment and cause harmful effects to non-target organisms. Nanoengineered materials, such as mesoporous silica nanocapsules (SiNCs) containing AF compounds, have been developed to control their release rate and reduce their toxicity to aquatic organisms. This study aimed to evaluate the acute toxicity of new nanoengineered materials, SiNC-DCOIT and a silver-coated form (SiNC-DCOIT-Ag), as well as the free form of DCOIT and empty nanocapsules (SiNCs), on the sun coral *Tubastraea coccinea*. *T. coccinea* is an invasive species and can be an alternative test organism for evaluating the risks to native species, as most native corals are currently threatened. The colonies were collected from the Alcatrazes Archipelago, SP, Brazil, and acclimatized to laboratory conditions. They were exposed for 96 h to different concentrations of the tested substances: 3.33, 10, 33, and 100 µg L^−1^ of free DCOIT; 500, 1000, 2000, and 4000 µg L^−1^ of SiNC; and 74.1, 222.2, 666.7, and 2000 µg L^−1^ of SiNC-DCOIT and SiNC-DCOIT-Ag. The test chambers consisted of 500 mL flasks containing the test solutions, and the tests were maintained under constant aeration, a constant temperature of 23 ± 2 °C, and photoperiod of 12 h:12 h (light/dark). At the end of the experiments, no lethal effect was observed; however, some sublethal effects were noticeable, such as the exposure of the skeleton in most of the concentrations and replicates, except for the controls, and embrittlement at higher concentrations. Adults of *T. coccinea* were considered slightly sensitive to the tested substances. This resistance may indicate a greater capacity for proliferation in the species, which is favored in substrates containing antifouling paints, to the detriment of the native species.

## 1. Introduction

Natural and human-built immersed structures are vulnerable to biofouling, which is the accumulation of various forms of aquatic organisms on such structural surfaces [1,2]. The increased friction caused by the hull of the colonized ship, associated with the weight gain due to biofouling, reduces the operational efficiency, increases costs, and threatens ship safety [2,3,4]. Biofouling may also favor bioinvasion, as has been reported for sun coral *Tubastraea* spp. across the Brazilian coast [5]. Bioinvasion results from the transport of allochthonous organisms and their establishment in regions where they would not occur based on natural dispersion, and often causes negative impacts, on the economic, social, and ecological levels.

Some strategies have been used to mitigate and manage biofouling, including the use of antifouling (AF) paints containing a range of chemical biocides in their composition. These paints have been used to cover ships, oil platforms, pipelines, and other submerged structures [3,6]. However, AF biocides are gradually leached from painted surfaces, causing environmental contamination. Some biocides may persist in the environment and induce adverse effects on non-target organisms [3,4,7].

The use of AF biocides has changed over time. First-generation AF compounds included copper and zinc oxides, but these had low durability and efficacy [7,8]. These compounds were replaced by organotin (OT)-based AFs, especially tributyltin (TBT), which was durable and effective against most fouling species [7,9,10]. However, because OTs were highly toxic to non-target organisms, bioaccumulative, and persistent, their use in AF coatings was banned globally by the International Maritime Organization after the International Convention on the Control of Harmful Anti-Fouling Systems on Ships [11]. The third generation of AF biocides was introduced in the market primarily from the mid 1990s, and it included organic compounds such as Diuron, Irgarol 1051, and 4,5-dichlorine-2-n-octyl-4-isothiazole-3-one, or DCOIT [1,12]. This new generation of AF biocides was designed to have three main characteristics: (i) be rapidly degradable; (ii) be toxic only to target organisms; and (iii) induce minimal bioconcentration [13].

DCOIT, also known as SeaNine, was proposed as an environmentally safe alternative to AF biocides [14,15], and authorized for use by the European Chemicals Agency (ECHA) and the United States Environmental Protection Agency (USEPA), based on its low level of global impact [1,3]. Some initial studies have suggested that DCOIT causes low environmental impacts, especially because of its rapid degradation (<24 h in natural seawater and <1 h in sediments) [6,15]. Thus, DCOIT has been reported to be one of the main biocides used in antifouling paints applied to maritime structures in recent years [1,3,10]. However, despite its short half life, DCOIT has been found in port areas worldwide [10,12] in concentrations potentially toxic to aquatic organisms. To manage this problem, new technologies to reduce the release of biocides to the water column and, consequently, reduce their impacts on non-target marine biota have been proposed [16], including the encapsulation of biocides in nanomaterials [4,17].

Biocide encapsulation prevents its direct interaction with the coating compounds and controls the leaching rates, increasing the coating durability and reducing surface colonization by various organisms [17]. Recently, two forms of nanocapsule containing DCOIT were developed, the first consisting of a silica nanocapsule (SiNC-DCOIT), and a second version in which SiNC was impregnated with silver (SiNC-DCOIT-Ag) [4]. Experiments comparing the antifouling efficacy and toxicity of free and nanoengineered forms of DCOIT in temperate organisms showed that SiNC-DCOIT and SiNC-DCOIT-Ag were less toxic than the free form [17,18]. Moreover, 11 marine species from temperate climates showed various levels of sensitivity to these new AF biocides, while SiNC-DCOIT-Ag reduced the toxicity and environmental danger to the species, without reducing the effectiveness of AF [17,18]. Similar results were observed in tropical mysids [4]. These studies suggested that nanoengineered materials containing DCOIT could be a suitable alternative to attenuate the effects of AF coatings. However, studies on polar and tropical species are necessary before the adoption of AF coatings that contain such substances can be recommended.

Shallow-water corals are the main ecosystems engineers in the tropics and are highly sensitive to pollution [19]. Here, we use the invasive sun coral *Tubastraea coccinea* (Lesson, 1829) as a model to investigate the toxicity of new nanoengineered antifouling, SiNC-DCOIT and SiNC-DCOIT-Ag, as well as the free form of DCOIT and empty nanocapsules (SiNCs). A previous study showed that *T. coccinea* exhibited adverse effects when exposed to high concentrations of some types of oils [20]. *T. coccinea* is an azooxanthellate species [21] from the Indo-Pacific introduced to the Brazilian coast via the opportunistic colonization of oil platforms and is currently recorded as existing along more than 3500 km of the coast, competing with native species [5,22,23,24]. *T. coccinea* is an interesting organism to use in toxicity assessments of antifouling compounds for tropical Brazilian environments, as its collection and use do not impact native ecosystems. This study aimed to evaluate the toxicity of antifouling DCOIT and its nanoengineered forms on the sun coral *T. coccinea* and observe its lethal and sublethal effects on animals.

## 2. Materials and Methods

### 2.1. Test Organism

*Tubastraea coccinea* Lesson, 1829, which was initially reported in Brazil in the 1980s, adheres to oil platforms [25,26]. Considered a cosmopolitan species, *T. coccinea* is commonly found along the Brazilian coast, covering both natural and artificial hard structures [5,22,27]. The rapid spread of *T. coccinea* across tropical seas is attributed to its high reproductive rates [28], occurrence of multiple events of introduction [22,29], early reproductive maturity [30], rapid growth and high recruitment rates [31], rare survival strategies [32], and notable regenerative capacity [5,33]. In Brazil, *T. coccinea* may serve as an alternative for assessing the impacts of pollutants on shallow-water corals, because most native species are threatened [34] and cannot be collected in the field for toxicity testing. In contrast, the removal of sun coral from the environment is encouraged by Brazilian environmental agencies. Nevertheless, we highlight that the toxicity of the antifouling compound might be species-specific, and we expect that the tolerance of native zooxanthellate species is likely lower.

### 2.2. Organisms Sampling

Approximately 250 colonies of *T. coccinea* were collected by scuba diving in the Alcatrazes Archipelago Wildlife Refuge in Southwestern Brazil (see Figure S1 in the Supplementary Materials), a federal marine protected area (MPA). Colonies were manually removed from the rocky reefs during removal operations organized by the authorities responsible for the MPA (ICMBio). The collections were authorized according to License SISBIO #79823-1.

**Figure 1:**
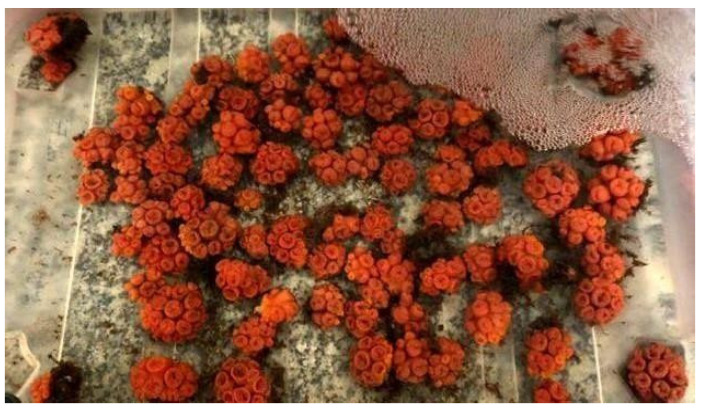
Colonies of *Tubastraea coccinea* during the acclimatization period in the laboratory. Source: the authors.

In the laboratory, the colonies were kept for five days in tanks with clean filtered sea water, under constant aeration, temperature of 23 ± 2 °C, and natural photoperiod (12 h:12 h light/dark) for acclimation (Figure 1). Water was partially renewed daily using reconstituted seawater (RedSea^®^, London, UK).

### 2.3. Characterization of Tested Compounds

Four substances were analyzed: (1) the biocide DCOIT; (2) the empty silica nanocapsules (SiNCs, acquired from Sigma-Aldrich, San Luis, MI, USA); (3) the nanomaterial containing the biocide SiNC-DCOIT; and (4) the nanomaterial containing the biocide SiNC-DCOIT-Ag. All substances were provided by Smallmatek Ltd. (Aveiro, Portugal). Stock solutions and dispersions were prepared for each substance from the dilution of salts in deionized water and dispersed in an ultrasonic bath (40 kHz) for 30 min. Then, the working solutions and dispersions were prepared by diluting the stock solutions in reconstituted seawater (salinity 34–35) followed by sonication for 15 min; these were immediately added to the replicates (500 mL glass flasks). The following concentrations were prepared for the test substances: 3.33, 10, 33, and 100 µg L^−1^ for free DCOIT; 500, 1000, 2000, and 4000 µg L^−1^ for SiNC; and 74.1, 222.2, 666.7, and 2000 µg L^−1^ for SiNC-DCOIT and SiNC-DCOIT-Ag (Table S1). The negative control consisted of reconstituted seawater (Red Sea^®^). These concentration ranges were chosen based on previous studies conducted on temperate marine species [17,18].

The tested nanomaterials were fully characterized by Figueiredo et al. [17] and Perina et al. [35]; concentrations were also confirmed by Perina et al. [35]. Briefly, SiNC has a core–shell and porous structure with a diameter of 129 nm, whereas SiNC-DCOIT has a diameter of 152 nm and a biocidal content of 18.3% [17,35]. Charged nanomaterials exhibit spherical and regular shapes. The hydrodynamic diameter obtained through dynamic light scattering (DLS) indicated that the size of the engineered nanomaterials (ENMs) varied between 202.67 and 231.11 nm. In turn, the determined polydispersity index (PdI) was within the range of 0.44–0.58, indicating average dispersity. The zeta potential (ζ) values in natural seawater were positive, and the dispersions tended to be unstable (30 mV > ζ > −30 mV), with the exception of SiNC at 0.001 µg L^−1^.

### 2.4. Toxicity Bioassay

The bioassays consisted of exposing the colonies of *T. coccinea* for 96 h to the test substances under constant conditions (23 ± 2 °C, natural photoperiod (12 h:12 h light/dark), and constant gentle aeration), as described in [20]. Colonies were tried and randomly introduced into 500 mL glass flasks containing the test solutions. Three replicates were prepared for each treatment. Physicochemical parameters of the test dilutions, such as dissolved oxygen (DO), pH, salinity, and temperature (T °C), were measured at the start and end of the experiments. At 96 h exposure, the colonies were inspected for visible alterations (such as tissue necrosis or endo-skeleton exposure) and counting of the number of dead polyps. 

### 2.5. Statistical Analyses

Results were first checked for normal distribution and homoscedasticity using the Shapiro–Wilk and Bartlett tests, respectively, and then analyzed by means of analysis of variance (ANOVA) followed by Dunnett’s test for multiple comparisons with the negative control (*p* < 0.05), or Kruskall–Wallis test followed by Dunn’s test, in the case of heterocedastic data or non-normal distribution. Statistical analyses were performed using Past 4.14 free software. In addition, Spearman’s non-parametric correlation test was used to check for significant relationships between the test concentrations and lethal and sub-lethal endpoints, as used in other studies [36,37].

## 3. Results

**Figure 2:**
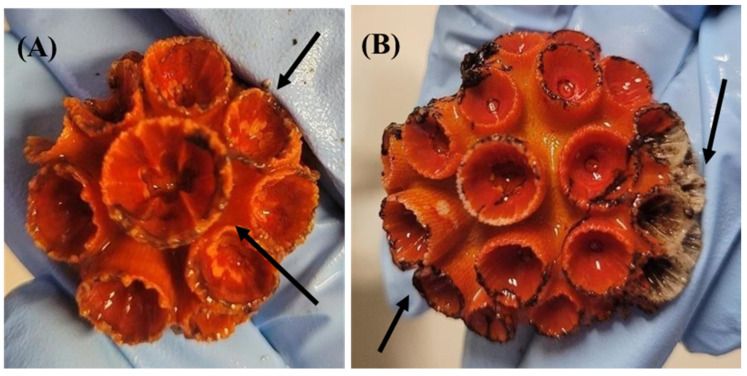
Sub-lethal effects observed in *Tubastraea coccinea* colonies exposed to antifouling biocides. (**A**) arrows show increased fragility of polyps and incipient tissue necrosis at 100 µg L^−1^ of DCOIT; (**B**) arrows show tissue necrosis and exposed polyps after tissue loss at 2000 µg L^−1^ of SiNC-DCOIT-Ag.

**Figure 3:**
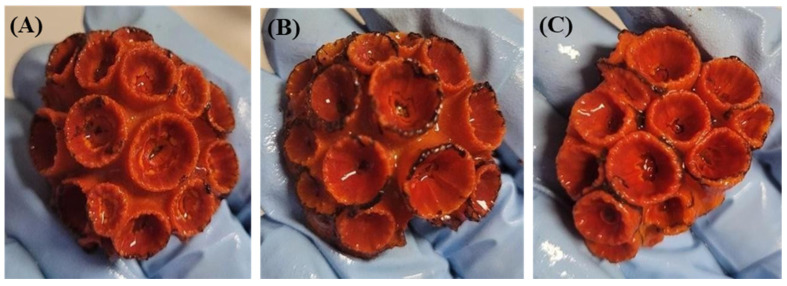
Appearance of the control colonies of *Tubastraea coccinea* at the end of the experiment. (**A**) Control 1; (**B**) Control 2; and (**C**) Control 3.

The results showed no significant lethal effects, since all colonies survived both the control and treatments (see Supplementary Materials for the raw results of the statistical analyses). Partial death was observed exclusively in colonies exposed to higher concentrations, affecting one and five polyps in the SiNC 4000 µg L^−1^ and SiNC-DCOIT-Ag 2000 µg L^−1^ treatments, respectively (Figure 2B; Table 1). Sub-lethal effects, such as tissue necrosis and initial signs of tissue loss leading to the exposure of the calcareous skeleton, were observed in all treatments and replicates (Figure 2A, Table 1), but the treatments did not differ from the control (for *p* = 0.05, see Supplementary Materials). Two replicates of the control showed initial signs of tissue necrosis, possibly due to manipulation stress (Figure 3). Skeleton fragility was observed exclusively in the organisms exposed to higher concentrations; in these conditions, polyps exhibited broken margins and dead polyps (Figure 2). However, there was no significant difference in the sub-lethal effects between the control and treatments. In addition, there were no significant correlations between the tested concentrations and lethal or sub-lethal responses. With regard to sub-lethal effects, when the results are compared qualitatively (i.e., presence or absence of effects), some trends can be detected. Fragile or dead polyps occurred only at the highest concentrations (i.e., 100 µg L^−1^ of DCOIT, 4000 µg L^−1^ of SiNC, 666.7 µg L^−1^ of SiNC-DCOIT, and 2000 µg L^−1^ of SiNC-DCOIT-Ag (Table 1)). The raw data are presented in Tables S2 and S3 (Supplementary Materials), while the raw results of the statistical analyses are shown in the SM as datasets.

## 4. Discussion

In this study, neither free DCOIT nor its nanostructured forms significantly affected *T. coccinea* polyps. This lack of severe effects might be explained by the rapid degradation of DCOIT under normal conditions [3,6,14,15], becoming low or non-toxic. However, DCOIT can be toxic to marine organisms before degradation occurs [4,35,38,39]. In addition, the nanostructured forms (i.e., SiNC-DCOIT and SiNC-DCOIT-Ag) were expected to be less toxic than the free DCOIT, as previously shown for other marine organisms from temperate regions [17,18]. Together, these results suggest a slow release of the nanostructured biocide into the water column and support the statement of Figueiredo et al. [17,18] that SiNC-DCOIT-Ag is a promising candidate for reducing the environmental impact of the third generation of booster biocides currently used, because of its lower toxicity and high efficiency as an AF biocide. Furthermore, Santos et al. [1] demonstrated that SiNC-DCOIT was less toxic than free DCOIT to larval stages of the brown mussel *Perna perna*, whereas Jesus et al. [4] found similar results for the mysid *Mysidopsis juniae*. Similarly, Campos et al. [40] observed that free DCOIT was more toxic to juveniles of the oyster *Crassostrea gigas* than to its nanostructured counterparts.

In our experiments, signs of sub-lethal effects were detected mainly at the highest concentrations of the tested substances, such as SiNC (4000 µg L^−1^), SiNC-DCOIT (666.7 µg L^−1^), and SiNC-DCOIT-Ag (2000 µg L^−1^), particularly using a qualitative analysis (i.e., presence/absence). However, some of these effects also occurred at lower concentrations, such as those observed for free DCOIT (100 µg L^−1^). Ferreira et al. [41] assessed the effects of free and nanostructured DCOIT on the symbiotic octocoral *Sarcophyton* cf. *glaucum* after a 7-day exposure and observed sublethal effects, such as coral polyp retraction, reduced photosynthetic efficiency, and increased levels of oxidative stress in organisms exposed to free DCOIT. Because our experiments evaluated the effects after short-term exposure, the occurrence of these sub-lethal effects may indicate the possibility of long-term effects; thus, further studies are required to assess the tolerance of *T. coccinea* during long-term exposure.

Antifouling biocides are often present in immersed anthropic structures, such as boats and pipelines, to provide protection against biofouling and avoid the establishment of biological communities on anthropic structures. As antifouling biocides, the tested substances were expected to inhibit and strongly intoxicate fouling organisms, as reported for marine temperate species [17,18] and juveniles of *P. perna* [1], but they seemed to not be as effective against adults of *T. coccinea*. Recently, Roepke et al. [42] studied the toxicity of DCOIT (free and encapsulated forms in cerium oxide nanoparticles) and observed that the antifouling inhibited algal fouling but did not affect coral larval settlement. Still, the lack of effects even at the highest concentrations (i.e., at magnitudes of milligrams per liter) shows that *T. coccinea* is highly tolerant to the AF biocides tested, another advantage aiding its spread along the Brazilian coast, including at seaports [43] and recreational marinas [44], where the concentration of antifouling biocides might be high. In this sense, the application of AF biocides would favor *T. coccinea* to the detriment of other species, and this may enhance the problem in areas where the sun coral is an invasive species, such as the Brazilian coast. Brockinton et al. [45] stated that anthropic factors could stimulate bioinvasion by sun corals; however, they did not mention the presence of immersed structures painted with antifouling coatings; thus, this factor should also be considered, including in the decommissioning of oil pipelines and platforms. Braga et al. [27] stated that the abandonment of oil platforms on the coast of the state of Ceará (Northeast Brazil) was the main factor that allowed the bioinvasion of the Brazilian North coast by *Tubastraea* spp. Despite the high tolerance of adult colonies, further studies using larval stages of *T. coccinea* are required, as these are the main targets of antifouling biocides.

## 5. Conclusions

A recent review showed that marine organisms may be vulnerable to AF biocides and recommended their substitution with environmentally friendly alternatives, such as nanoengineered forms [10]. Our study shows that DCOIT, in its free and nanostructured forms, did not cause lethal effects to *T. coccinea* adults, even at higher concentrations. No significant sub-lethal effects occurred, but at the highest concentrations, some colonies displayed incipient qualitative effects after 96 h of exposure. Thus, *T. coccinea* is tolerant to the tested biocides, at least in the short term, which may provide this species with a stronger ability to disperse and colonize submerged structures, including those painted with antifouling coatings.

## Figures and Tables

**Table 1:** Lethal and sub-lethal effects observed in *Tubastraea coccinea* exposed to new antifouling substances, at each concentration.

Treatment	Concentration (µg L^−1^)	Tissue Necrosis (% of Total Colonies/Replicate)	Fragile Polyps (n.)	Dead Polyps (n.)
Control 1	0	*	0	0
Control 2	0	*	0	0
Control 3	0	*	0	0
DCOIT	3.33	100	0	0
	10	66	0	0
	33	100	0	0
	100	66	2	0
SiNC	500	66	0	0
	1000	33	1	0
	2000	33	0	0
	4000	33	2	1
SiNC-DCOIT	74.1	100	0	0
	222.2	100	0	0
	666.7	33	3	0
	2000	33	0	0
SiNC-DCOIT-Ag	74.1	100	0	0
	222.2	66	1	0
	666.7	66	0	0
	2000	100	2	5

DCOIT = 4,5-dichlorine-2-n-octyl-4-isothiazole-3-one; SiNCs = silica nanocapsules (empty); SiNC-DCOIT = silica nanocapsules charged with 4,5-dichlorine-2-n-octyl-4-isothiazole-3-one; SiNC-DCOIT-Ag = silica nanocapsules charged with 4,5-dichlorine-2-n-octyl-4-isothiazole-3-one and coated with silver (Ag). * For Controls 1 and 2, the images suggest an incipient tissue loss in some polyps, but not significant for the colony. In Control 3, all the polyps look healthy.

## Data Availability

All the results of this investigation are available in the manuscript or in the respective supplementary materials.

## Supplementary Materials

The following supporting information can be downloaded at: https://www.mdpi.com/article/10.3390/toxics12010044/s1, Figure S1: Map showing the sampling site of colonies of *Tubastraea coccínea* (sun coral) in the Alcatrazes Archipelago, SP, Brazil. Source: Google Earth (2022); Table S1: Concentration of the test substances during the experiments with antifouling biocides and the sun coral *Tubastraea coccinea*; Table S2. Physicochemical parameters of test solutions measured at the start and the end of the experiments with *Tubastraea coccinea* and new antifouling biocides; Table S3. Raw data showing the effects of new antifouling biocides on colonies of *Tubastraea coccinea*, for each experimental replicate.
