# Supplementary material for: Adults of Sun Coral Tubastraea coccinea (Lesson 1829) Are Resistant to New Antifouling Biocides
Source: Toxics. 2024 Jan 6;12(1):44. doi: 10.3390/toxics12010044 (PMC10818711; doi:10.3390/toxics12010044)
Supplement: Supplementary file 1 [file toxics-12-00044-s001.zip › toxics-2800443-supplementary.pdf]

toxics

MDPI

Supplementary Materials: Adults of Sun Coral *Tubastraea coccinea* (Lesson 1829) Are Resistant to New Antifouling Biocides

Isabela Martins, Kátia Cristina Cruz Capel and Denis Moledo de Souza Abessa

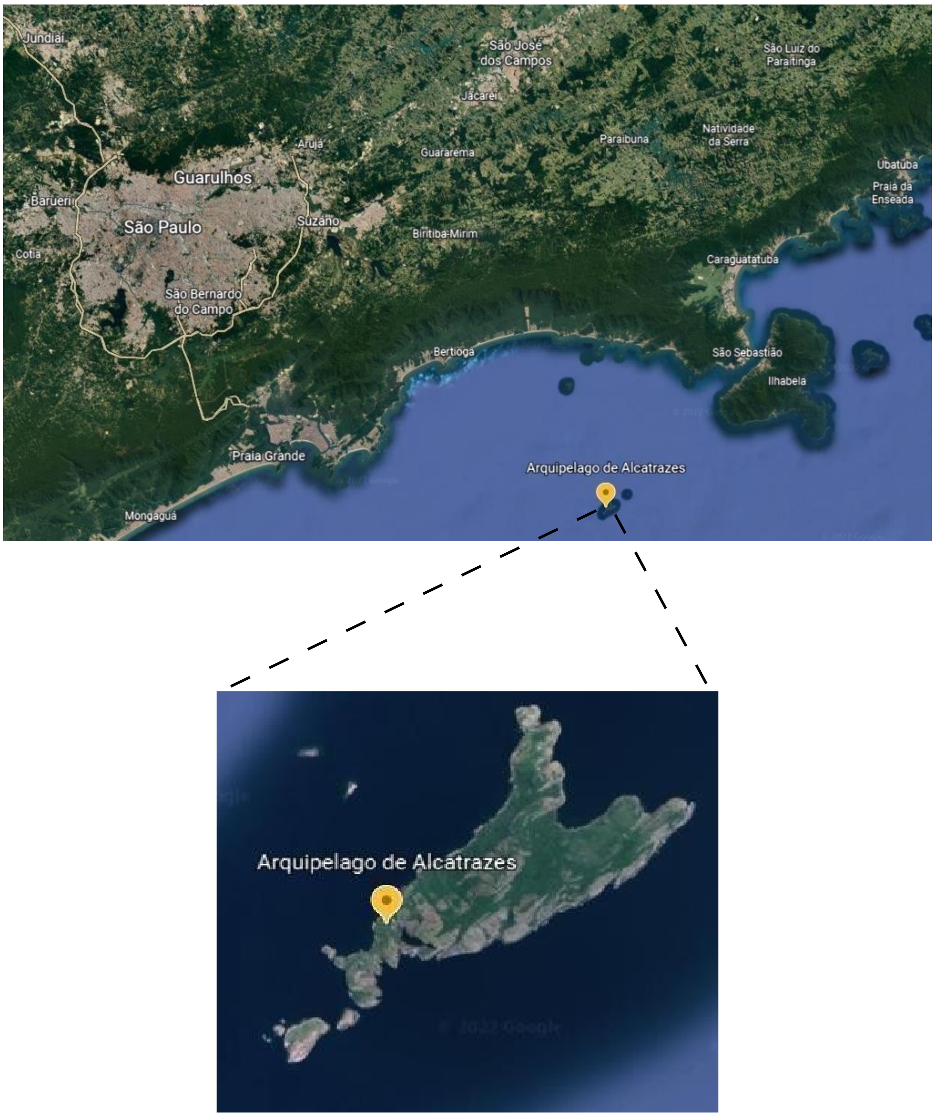

Figure S1. Map showing the sampling site of colonies of *Tubastraea coccínea* (sun-coral) in the Alcatrazes Archipelago, SP, Brazil. Source: Google Earth (2022).

Table S1. Concentration of the test-substances during the experiments with antifouling biocides and the sun-coral *Tubastraea coccinea*.

Substance	Concentrations (µg L^{-1})
DCOIT	3.33
	10
	33.3
	100
SiNC	500
	1000
	2000
	4000
SiNC-DCOIT	74.1
	222.2
	666.7
	2000
SiNC-DCOIT-Ag	74.1
	222.2
	666.7
	2000

DCOIT = 4,5-dichlorine-2-n-octyl-4-isothiazole-3-one; SiNC = Silica nanocapsules (empty); SiNC-DCOIT = Silica nanocapsules charged with 4,5-dichlorine-2-n-octyl-4-isothiazole-3-one; SiNC-DCOIT-Ag = Silica nanocapsules charged with 4,5-dichlorine-2-n-octyl-4-isothiazole-3-one and coated with silver (Ag).

Copyright: © 2024 by the authors. Licensee MDPI, Basel, Switzerland. This article is an open access article distributed under the terms and conditions of the Creative Commons Attribution (CC BY) license (https://creativecommons.org/licenses/by/4.0/).

Table S2. Physicochemical parameters of test-solutions measured in the start and the end of the experiments with *Tubastraea coccinea* and new antifouling biocides. (DCOIT = 4,5-dichlorine-2-n-octyl-4-isothiazole-3-one; SiNC = Silica nanocapsules (empty); SiNC-DCOIT = Silica nanocapsules charged with 4,5-dichlorine-2-n-octyl-4-isothiazole-3-one; SiNC-DCOIT-Ag = Silica nanocapsules charged with 4,5-dichlorine-2-n-octyl-4-isothiazole-3-one and coated with silver (Ag)).

Treatment	Concentration (µg L^{-1})	Replicate (n.)	pH		T (°C)		D.O. (%)	
			initial	final	initial	final	initial	final
Control	-	1	7.79	7.90	23.5	23.2	79.7	88.7
	-	2	7.79	7.97	23.5	23.2	80.1	87.8
	-	3	7.74	7.89	23.5	23.2	79.3	87.0
DCOIT	3.33	1	7.66	7.96	23.7	22.7	83.3	89.2
	10	1	7.56	7.82	23.7	23.1	77.9	93.4
	33	3	7.57	7.69	23.8	22.8	83.8	94.3
	100	3	7.65	7.99	24.0	23.2	80.1	97.6
SiNC	500	1	7.92	7.97	24.4	23.8	91.7	97.5
	1000	1	7.94	8.01	23.5	23.2	83.8	94.4
	2000	2	7.97	8.03	24.1	23.3	89.6	94.4
	4000	1	7.82	7.98	24.2	23.3	83.9	91.9
SiNC-DCOIT	74.1	1	7.61	7.90	23.9	23.8	75.5	86.1

	222.2	2	7.83	7.85	23.4	23.6	81.1	88.0
	666.7	1	7.76	7.89	24.1	23.7	83.5	83.8
	2000	1	7.88	7.98	23.6	23.8	84.5	92.6
SiNC-DCOIT-Ag	74.1	1	7.86	7.99	23.3	23.5	87.7	88.4
	222.2	3	7.88	7.99	23.3	23.3	82.0	87.4
	666.7	2	7.81	8.02	23.4	23.5	81.9	89.1
	2000	3	7.88	7.99	23.4	23.4	85.5	88.7

Table S3. Raw data regarding the effects of new antifouling biocides on colonies of *Tubastraea coccinea,* for each experimental replicate.

Treatment	Concentration (μg L^{-1})	Replicate (n.)	Total ofpolyps (n.)	Tissue loss / polyp (n.)	Algae on polyps margins (Presence/Absence)
Control	-	1	19	3	Absent
	-	2	17	6	Present
	-	3	14	0	Present
DCOIT	3.33	1	14	1	Absent
		2	17	7	Present
		3	15	3	Present
	10	1	13	6	Present
		2	15	3	Present
		3	16	6	Absent
	33	1	13	2	Absent
		2	12	3	Present
		3	12	5	Present
	100	1	13	3	Present
		2	20	3	Absent
		3	11	5	Present
SiNC	500	1	8	0	Absent
		2	17	0	Absent
		3	12	0	Absent
	1000	1	9	0	Absent
		2	12	0	Absent
		3	13	0	Absent
	2000	1	10	0	Absent
		2	7	0	Absent
		3	11	0	Absent
	4000	1	11	0	Absent
		2	14	0	Absent
		3	12	0	Absent
SiNC-DCOIT	74.1	1	13	2	Present
		2	18	3	Present
		3	15	3	Absent
	222.2	1	15	7	Present
		2	15	8	Present
		3	11	6	Present
	666.7	1	10	2	Absent
		2	12	0	Absent
		3	11	0	Absent
	2000	1	9	0	Absent
		2	8	0	Absent
		3	10	1	Absent
SiNC-DCOIT-Ag	74.1	1	16	4	Present
		2	19	8	Present
		3	21	6	Present

	1	15	6	Present
222,2	2	13	8	Present
	3	14	1	Absent
	1	17	5	Absent
666.7	2	20	20	Present
	3	20	18	Present
	1	19	7	Present
2000	2	12	3	Present
	3	20	5	Absent

DCOIT = 4,5-dichlorine-2-n-octyl-4-isothiazole-3-one; SiNC = Silica nanocapsules (empty); SiNC-DCOIT = Silica nanocapsules charged with 4,5-dichlorine-2-n-octyl-4-isothiazole-3-one; SiNC-DCOIT-Ag = Silica nanocapsules charged with 4,5-dichlorine-2-n-octyl-4-isothiazole-3-one and coated with silver (Ag).

Raw results of statistical analyses – Mortality

<u>DCOIT (Non parametric Kruskall-Wallis test)</u>

H (chi2): 0

Hc (tie corrected): NAN

p (same): NAN

There is no significant difference between sample medians

<u>SiNC (Non parametric Kruskall-Wallis test)</u>

H (chi2): 0,375

Hc (tie corrected): 2

p (same): 0,3679

There is no significant difference between sample medians

<u>SiNC-DCOIT (Non parametric Kruskall-Wallis test)</u>

H (chi2): 0

Hc (tie corrected): NAN

p (same): NAN

There is no significant difference between sample medians

<u>SiNC-DCOIT-Ag (Non parametric Kruskall-Wallis test)</u>

H (chi2): 0,38

Hc (tie corrected): 1,086

p (same): 0,5811

There is no significant difference between sample medians

Raw results of statistical analyses – Broken polyps

DCOIT (Non parametric Kruskall-Wallis test)

H (chi2): 0,375

Hc (tie corrected): 2

p (same): 0,3679

There is no significant difference between sample medians

SiNC (Non parametric Kruskall-Wallis test)

H (chi2): 0,38

Hc (tie corrected): 1,086

p (same): 0,5811

There is no significant difference between sample medians

SiNC-DCOIT (Non parametric Kruskall-Wallis test)

H (chi2): 0,38

Hc (tie corrected): 1,086

p (same): 0,5811

There is no significant difference between sample medians

SiNC-DCOIT-Ag (Non parametric Kruskall-Wallis test)

H (chi2): 0,015

Hc (tie corrected): 0,03077

p (same): 0,9847

There is no significant difference between sample medians

Raw results of statistical analyses – Loss of tissues of polyps

DCOIT (One way ANOVA)

Test for equal means

	Sum of sqrs	df	Mean square	F	p (same)
Between groups:	1461,64	3	487,213	0,7011	0,5693
Within groups:	8338,86	12	694,905	Permutation p (n=99999)	
Total:	9800,5	15	0,9233		

Components of variance (only for random effects):

Var(group): -51,923 Var(error): 694,905 ICC: -0,0807534

omega2: 0

Levene´s test for homogeneity of variance, from means p (same): 0,008164

Levene´s test, from medians p (same): 0,5669

Welch F test in the case of unequal variances: F=0,584, df=5,33, p=0,6496

Bayes factor: 0,1828 (substantial evidence for equal means)

SiNC (Non parametric Kruskall-Wallis test)

H (chi2): 0,05515

Hc (tie corrected): 0,09518

p (same): 0,9924

There is no significant difference between sample medians

SiNC-DCOIT (One way ANOVA)

Test for equal means

	Sum of sqrs	df	Mean square	F	p (same)
Between groups:	575609	3	191870	0,6824	0,5797
Within groups:	3,37416E06	12	281180	Permutation p (n=99999)	
Total:	3,94977E06	15	0,9985		

Components of variance (only for random effects):

Var(group): -22327,5 Var(error): 281180 ICC: -0,0862558

omega2: 0

Levene´s test for homogeneity of variance, from means p (same): 0,00894

Levene´s test, from medians p (same): 0,5791

Welch F test in the case of unequal variances: F=0,5484, df=5,357, p=0,6694

Bayes factor: 0,1793 (substantial evidence for equal means)

SiNC-DCOIT-Ag (One way ANOVA)

Test for equal means

	Sum of sqrs	df	Mean square	F	p (same)
Between groups:	0,538597	4	0,134649	2,54	0,1057
Within groups:	0,530014	10	0,0530014	Permutation p (n=99999)	
Total:	1,06861	14	0,09885		

Components of variance (only for random effects):

Var(group): 0,027216 Var(error): 0,0530014 ICC: 0,339278

omega2: 0,2912

Levene´s test for homogeneity of variance, from means p (same): 0,06967

Levene´s test, from medians p (same): 0,6295

Welch F test in the case of unequal variances: F=1,016, df=4,691, p=0,4832

Bayes factor: 0,9736 (no evidence for either equal or unequal means)

Disclaimer/Publisher's Note: The statements, opinions and data contained in all publications are solely those of the individual author(s) and contributor(s) and not of MDPI and/or the editor(s). MDPI and/or the editor(s) disclaim responsibility for any injury to people or property resulting from any ideas, methods, instructions or products referred to in the content.
